# Vascular compliance phenotyping in pulmonary arterial hypertension (a PVDOMICS study)

**DOI:** 10.1016/j.jhlto.2026.100560

**Published:** 2026-04-11

**Authors:** Talitha G. Wilson, Laura Oppegard, Hongyang Pi, Brian A. Houston, W.H. Wilson Tang, Evelyn M. Horn, Monica Mukherjee, Peter J. Leary, Jeffrey C. Robinson

**Affiliations:** a Department of Pulmonary, Allergy, and Critical Care Medicine, Oregon Health and Science University, Portland, OR; b University of Southern California Los Angeles General Medical Center, Los Angeles, CA; c Pulmonary and Critical Care Medicine, University of Washington, Seattle, WA; d Department of Medicine, Division of Cardiology, Medical University of South Carolina, Charleston, SC; e Department of Cardiovascular Medicine, Cleveland Clinic, Cleveland, OH; f Department of Medicine, Division of Cardiology, Weill-Cornell Medical School, New York, NY; g Division of Cardiology, Johns Hopkins University School of Medicine, Baltimore, MD; h Department of Epidemiology, University of Washington, Seattle, WA

**Keywords:** Right heart failure, Cardiac adaptation, Hemodynamics, Connective tissue disease, Pulmonary arterial hypertension

## Abstract

**Background:**

Pulmonary arterial compliance is reduced in pulmonary arterial hypertension and may be a valuable prognostic marker; however, little is known about phenotypic factors or how compliance correlates with a range of outcomes. Our objective was to identify factors associated with compliance and evaluate relationships between compliance, cardiac morphology, exercise metrics, and mortality for individuals with pulmonary arterial hypertension and otherwise similar resistive afterload.

**Methods:**

We analyzed data from adult participants of the PVDOMICS cohort with World Symposium Group 1 pulmonary arterial hypertension. Compliance was derived by right heart catheterization measurement of stroke volume divided by pulmonary artery pulse pressure. Linear regression and Cox proportional hazards were used to estimate associations with outcomes in staged models.

**Results:**

In the cohort study population of 328 participants, several factors were associated with worse pulmonary arterial compliance, including older age, shorter time since diagnosis, higher pulmonary arterial wedge pressure, and connective tissue disease-associated pulmonary arterial hypertension. These associations persisted among individuals with otherwise similar pulmonary vascular resistance. Compliance was also associated with cardiac magnetic resonance-derived right ventricular morphology and exercise tolerance. There was a protective association between compliance and death or transplant, particularly among those with a mean pulmonary artery pressure ≤40 mmHg (HR = 0.44 per 1 mL/mmHg better compliance [95% CI 0.24, 0.82], *p* = 0.009).

**Conclusions:**

Pulmonary arterial compliance is associated with several patient factors, right heart adaptation, exercise tolerance, and survival. Future studies are needed to investigate the role of compliance in the pathophysiology of connective tissue disease-associated pulmonary arterial hypertension, risk stratification, and endotypes of disease.

## Background

Pulmonary arterial hypertension (PAH) results from pulmonary vascular remodeling leading to obstruction of small pulmonary arterioles. These changes increase right ventricular (RV) afterload, resulting in RV failure and death. Contributors to right heart afterload are complex but may be conceptually summarized by the 3-component Windkessel model.^1^ This model suggests that RV afterload includes contributions from pulmonary arterial compliance (PAC), pulmonary vascular resistance (PVR), and characteristic impedance, with PVR and PAC being the predominant contributors. PVR can be thought of as “static” RV afterload, and PAC, which incorporates distensibility and recoil, accounts for the pulsatile contribution to RV afterload.^2^

PVR has long been evaluated in pulmonary hypertension (PH) as a surrogate for total RV afterload using conventional right heart hemodynamic parameters; however, this focus on static afterload has yielded mixed prognostic power and limited ability to differentiate disease features, including symptom burden and right heart morphology.^3^, ^4^, ^5^ PAC is an expanding area of interest in PAH and may yield more accurate prognostication than PVR.^6^, ^7^ Novel studies of pulsatile afterload have demonstrated that failure to improve PAC after PAH-directed therapy is a poor prognostic factor, and PAC is an independent predictor of mortality.^6^, ^8^, ^9^

The relationship between PVR and PAC and their independent impacts on outcomes including right heart structure and function are areas of ongoing research. Prior work suggests an inverse-hyperbolic relationship between PVR and PAC with a constant resistance times compliance product, referred to as RC time.^10^, ^11^ This informed the previous paradigm of using PVR as a surrogate for total right heart afterload.^12^ In contrast, other studies suggest the correlation between PVR and PAC may be more variable, and each may have distinct contributions to right heart afterload, particularly when PVR is borderline or low.^13^, ^14^, ^15^ Whether PAC independently affects outcomes in PAH patients with similar PVR remains unclear.^16^, ^17^

In the current work, we used the PVDOMICS (Redefining Pulmonary Hypertension through Pulmonary Vascular Disease Phenomics) cohort to identify factors associated with PAC and evaluate relationships between compliance, cardiac morphology, exercise metrics, and mortality. Given the possibility that PAC and PVR are highly correlated, we evaluated whether associations with compliance persisted among individuals with otherwise similar PVR. A central hypothesis is that variable stiffness and PAC may contribute to residual differences in right heart afterload that are not fully accounted for by PVR.

## Materials and Methods

### Cohort

We evaluated participants in the PVDOMICS cohort, a multicenter observational study of patients with PH.^18^ Participants were enrolled between November 2016 and October 2019 at 7 centers across the United States. Eligibility criteria included age ≥18 years old, World Symposium Group 1 PAH, and completed the phenotyping protocol.^18^, ^19^ An assignment of Group 1 PAH could be suggested by the site; however, the cohort used in this analysis was the slightly smaller group of participants felt to have Group 1 PAH following expert adjudication by a centralized PVDOMICS committee. Local institutional review boards approved the parent study, and all participants provided informed consent. The PVDOMICS steering committee approved the proposal in October 2023, and the University of Washington review board determined that the study was exempt from further approval (UW HSD #19058).

### Exposures, outcomes, and covariates

The primary exposure of interest was PAC. Compliance was calculated from stroke volume (SV) divided by pulmonary artery pulse pressure (PApp) derived by right heart catheterization (RHC). RHC was performed during the baseline exam as part of the study protocol along with assessment of cardiac morphology and exercise testing for participants involved in these testing domains. All hemodynamic tracings were adjudicated and reviewed by a central hemodynamic core laboratory for assurance of data quality and accuracy.^19^

Outcomes of interest included cardiac morphology, exercise metrics, and mortality or transplant. Cardiac morphology was assessed using cardiac magnetic resonance (CMR) and analyzed centrally by the MRI Core lab for a subset of PVDOMICS participants. Exercise performance was determined using cardiopulmonary exercise testing (CPET) using standard protocols in a subset of participants who received CMR imaging. Gas exchange was evaluated using diffusion capacity for carbon monoxide (DLCO). Participants were followed for transplant and all-cause mortality by contact at least annually up to 10 years after enrollment.^18^

Covariates included age, sex, height, BMI, time since diagnosis (self-reported at the baseline examination), etiology of PAH (as adjudicated by PVDOMICS investigators and including idiopathic/familial [IPAH/fPAH], drug/toxin associated, connective tissue disease associated [CTD-PAH], or unknown/other), pulmonary artery wedge pressure (PAWP), and PVR. PAWP and all invasive hemodynamics were measured at end-exhalation during normal spontaneous breathing.

### Statistical analysis

Participant characteristics were presented using descriptive measures. Linear regression was used to evaluate associations between patient-level factors and PAC. An additional model was adjusted by PVR to evaluate whether these characteristics remained independently associated with compliance among individuals with otherwise similar resistance. A graphical representation of the relationship was shown by plotting the relationship of PVR to PAC with a best-fit line, with characteristics of interest dichotomized around the median value.

Subsequent analyses used linear regression in staged pre-specified models to evaluate the relationship between PAC, cardiac morphology, exercise metrics and DLCO. Cox proportional hazards were used to estimate the association between PAC and death or transplant and used a similar adjustment approach.

Exploratory analyses considered whether relationships between PAC and outcomes were distinct for different absolute levels of mean pulmonary arterial pressure (mPAP). In this approach, known as an isobaric comparison, models were adjusted for mPAP to evaluate whether relationships were dependent on pressure.^14^ These analyses are included in the supplement. Analyses were performed using Stata statistical software (STATA 18.0/SE), and a *p*-value of <0.05 was used to suggest significance.

## Results

**Figure 1:**
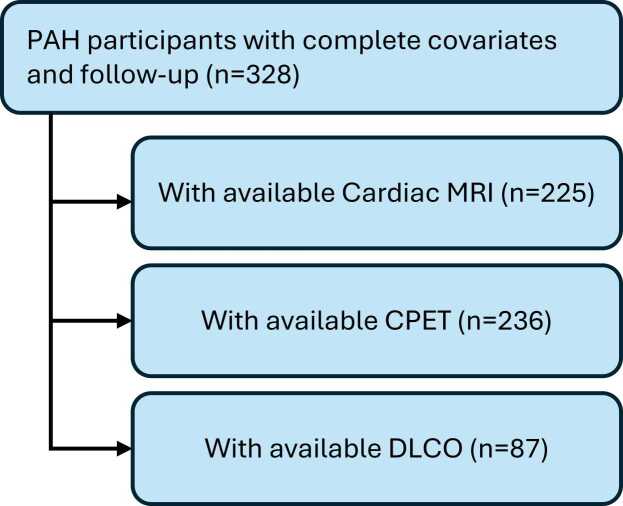
PVDOMICS participants included in the analyses.

There were 328 participants with complete data available for analyses of patient-level factors associated with PAC and the relationship of PAC with mortality or transplant. Of these participants, 225 participants had complete CMR data available and were included in CMR analyses, 236 participants had complete CPET data available and were included in exercise analyses, and 87 had DLCO available and were included in DLCO analyses (Figure 1). On average, participants were 52.7 ± 14.3 years old, 73.6% were women, and BMI was 29.1 ± 7.5 kg/m^2^. PAH etiology was most commonly idiopathic or heritable (53.6%), followed by connective tissue disease (28.7%), drug or toxin-related (4.8%), or other/unknown (12.9%). The median mPAP was 43 mmHg (Interquartile range (IQR) 33-54 mmHg), PAWP 10 mmHg (IQR 7-13mmHg), and PVR 6.2 WU (IQR 4.0-9.4 WU).

### Association between compliance and participant characteristics

**Figure 2:**
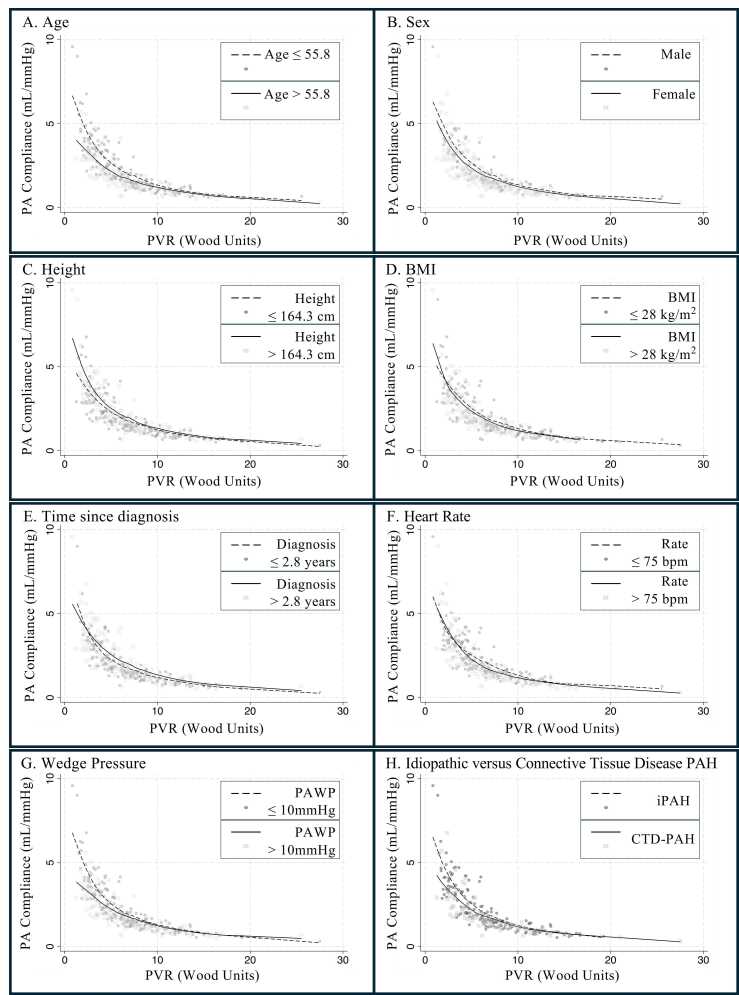
The relationship of pulmonary vascular resistance (PVR) to compliance stratified by characteristics of interest, including (A) age, (B) sex, (C) height, (D) body mass index (BMI), (E) time since diagnosis, (F) heart rate, (G) pulmonary arterial wedge pressure, and (H) idiopathic pulmonary arterial hypertension (iPAH) vs connective tissue disease-associated pulmonary arterial hypertension (CTD-PAH). Continuous variables stratified by median.

Several characteristics were associated with worse compliance (older age, shorter height, shorter time since diagnosis of PH, faster heart rate, higher PAWP, and CTD-associated PAH) (Table 1). The impression of an association persisted or strengthened after accounting for differences in PVR, with the exception of heart rate, which was no longer significant. In addition, there was a suggestion that a history of systemic hypertension was associated with worse compliance among individuals of otherwise similar PVR that was not seen prior to adjustment. Visual separation between variables of interest was apparent when dichotomized around the median (most notable with age, PAWP, and CTD-PAH relative to IPAH) (Figure 2). In these cases, the divergence between groups was most notable among individuals with lower PVR. Additional post-hoc exploratory analyses of the association between PAC and CTD-PAH accounting for a range of potential confounders did not fundamentally change the impression of lower PAC for a given PVR among participants with CTD-PAH (Supplemental Table 1).

**Table 1:** In Patients With PAH, Difference in Pulmonary Arterial Compliance by Participant Characteristic in Unadjusted Models and Among Individuals of Otherwise Similar Pulmonary Vascular Resistance (PVR) as Evaluated by Adjustment (Negative Compliance is Worse Compliance and Positive Compliance is Better Compliance) (*n* = 328)

	Difference in compliance			Difference in compliance		
	(Unadjusted)			(Individuals with similar PVR)		
	β	95% CI	*p*-value	β	95% CI	*p*-value
Age (per 10 years)	−0.14	(−0.26, −0.02)	0.02	−0.20	(−0.29, −0.10)	<0.001
Female	−0.07	(−0.46, 0.32)	0.72	−0.17	(−0.48, 0.13)	0.27
Height (per 10 cm)	0.24	(0.07, 0.42)	0.006	0.17	(0.03, 0.31)	0.01
BMI (per 5 kg/m^2^)	0.08	(−0.04, 0.19)	0.19	−0.06	(−0.15, 0.04)	0.23
Time since diagnosis (per 3 years)	0.13	(0.04, 0.22)	0.003	0.12	(0.06, 0.19)	<0.001
Heart rate (per 10 beats/min)	−0.26	(−0.38, −0.13)	<0.001	−0.07	(−0.17, 0.04)	0.21
PAWP (per 1 mmHg)	−0.04	(−0.07, −0.01)	0.01	−0.21	(−0.23, −0.18)	<0.001
History of hypertension	−0.27	(−0.64, 0.09)	0.14	−0.33	(−0.62, −0.04)	0.02
History of diabetes	−0.07	(−0.52, 0.39)	0.77	−0.26	(−0.62, 0.09)	0.15
PAH etiology						
Idiopathic		Referent			Referent	
Drug/toxin	−0.57	(−1.42, 0.28)	0.19	−0.42	(−1.09, 0.26)	0.22
Connective tissue disease	−0.52	(−0.94, −0.11)	0.01	−0.40	(−0.73, −0.06)	0.02
Other	−0.31	(−0.87, 0.26)	0.29	0.03	(−0.42, 0.49)	0.89

Abbreviations: BMI, body mass index; PAH, Pulmonary arterial hypertension; PAWP, pulmonary artery wedge pressure.

Compliance was also analyzed among individuals of otherwise similar mPAP (Supplemental Table 2). Trends were broadly similar among individuals with an otherwise similar mPAP.

### Cardiac morphology and PAC

After accounting for differences in body habitus, PAC was associated with all aspects of CMR-derived morphology (Table 2). Improved compliance was associated with a smaller right atrial end diastolic volume (RA-EDV), a smaller RV end diastolic volume (RV-EDV), a smaller RV mass, and a higher RV ejection fraction. None of these associations were significant after adjusting for PVR, suggesting that associations between compliance and cardiac morphology are not independent of differences in resistive afterload. Similar to what was seen with PVR adjustment, relationships between compliance and cardiac morphology were markedly attenuated when accounting for differences in mPAP (Supplemental Table 3).

**Table 2:** In Patients With PAH, Difference in Cardiac Morphology Relative to Pulmonary Arterial Compliance Accounting for Differences in Body Habitus (Height and Weight), Age, Sex, and Pulmonary Vascular Resistance (PVR) in Staged Models (*n* = 225)

	Difference in outcome per 1mL/mmHg better compliance		
	β	95% CI	*p*-value
RV-EDV (mL)			
Unadjusted	−5.2	(−11.8, 1.4)	0.12
Adjusted for body habitus (height and weight)	−7.1	(−13.3, −0.9)	0.03
Adjusted for age, sex, PAH etiology, and body habitus	−7.3	(−13.6, −1.0)	0.02
Adjusted for age, sex, PAH etiology, body habitus, and PVR	−1.9	(−10.1, 6.4)	0.66
			
RA-EDV (mL)			
Unadjusted	−4.7	(−7.3, −2.0)	0.001
Adjusted for body habitus (height and weight)	−5.2	(−7.8, −2.6)	<0.001
Adjusted for age, sex, PAH etiology, and body habitus	−5.1	(−7.7, −2.4)	<0.001
Adjusted for age, sex, PAH etiology, body habitus, and PVR	−1.1	(−4.5, 2.4)	0.54
			
RV-Mass (g)			
Unadjusted	−1.7	(−3.2, −0.1)	0.04
Adjusted for body habitus (height and weight)	−2.1	(−3.6, −0.6)	0.005
Adjusted for age, sex, PAH etiology, and body habitus	−2.1	(−3.6, −0.6)	0.005
Adjusted for age, sex, PAH etiology, body habitus, and PVR	0.4	(−1.5, 2.3)	0.65
			
RV-EF (%)			
Unadjusted	2.7%	(1.8%, 3.6%)	<0.001
Adjusted for body habitus (height and weight)	2.9%	(2.0%, 3.7%)	<0.001
Adjusted for age, sex, PAH etiology, and body habitus	2.9%	(2.0%, 3.9%)	<0.001
Adjusted for age, sex, PAH etiology, body habitus, and PVR	0.5%	(−0.6%, 1.6%)	0.41

Abbreviations: PAH, Pulmonary arterial hypertension; RA-EDV, Right atrial end diastolic volume; RV, Right ventricle; RV-EDV, Right ventricular end diastolic volume

### Exercise metrics and PAC

Individuals with higher PAC had significantly better exercise tolerance with higher maximal oxygen consumption, higher maximal achieved watts, and higher maximal metabolic equivalent of task (METs) (Table 3). This relationship was not dependent on body habitus, age, sex, or PAH etiology. In contrast to the association with cardiac morphology, the association between PAC and exercise tolerance was independent of PVR. Even among individuals with similar PVR, increased PAC was associated with a higher maximal oxygen consumption, higher maximal achieved watts, and more METs (Table 3). These associations with improved compliance also remained significant after accounting for mPAP (Supplemental Table 4). In the smaller subset of participants with DLCO, there was no statistically significant association between PAC and DLCO (Table 3).

**Table 3:** In Patients With PAH, Difference in Exercise and the Diffusing Capacity of the Lungs for Carbon Monoxide (DLCO) Relative to Pulmonary Arterial Compliance Accounting for Differences in Body Habitus (Height and Weight), Age, Sex, and Pulmonary Vascular Resistance (PVR) in Staged Models

	Difference in outcome per 1mL/mmHg better compliance		
	β	95% CI	*p*-value
Maximal oxygen consumption (mL/kg/min) (*n* = 236)			
Unadjusted	1.2	(0.9, 1.5)	<0.001
Adjusted for height (oxygen consumption already indexed to weight)	1.1	(0.8, 1.4)	<0.001
Adjusted for age, sex, PAH etiology, and height	1.1	(0.8, 1.4)	<0.001
Adjusted for age, sex, PAH etiology, height, and PVR	1.1	(0.7, 1.4)	<0.001
			
Maximal Watts (*n* = 236)			
Unadjusted	9.4	(6.6, 12.1)	<0.001
Adjusted for body habitus (height and weight)	8.1	(5.4, 10.8)	<0.001
Adjusted for age, sex, PAH etiology, and body habitus	7.4	(4.8, 9.7)	<0.001
Adjusted for age, sex, PAH etiology, body habitus, and PVR	5.7	(2.6, 8.9)	<0.001
Maximal Mets (*n* = 236)			
			
Unadjusted	0.3	(0.2, 0.4)	<0.001
Adjusted for body habitus (height and weight)	0.3	(0.2, 0.4)	<0.001
Adjusted for age, sex, PAH etiology, and body habitus	0.3	(0.2, 0.4)	<0.001
Adjusted for age, sex, PAH etiology, body habitus, and PVR	0.3	(0.2, 0.4)	<0.001
			
DLCO (*n* = 87)			
Unadjusted	1.1	(0.3, 1.9)	0.006
Adjusted for body habitus (height and weight)	0.3	(−0.4, 1.1)	0.35
Adjusted for age, sex, PAH etiology, and body habitus	0.4	(−0.2, 1.0)	0.15
Adjusted for age, sex, PAH etiology, body habitus, and PVR	0.5	(−0.3, 1.3)	0.24

Abbreviation: PAH, Pulmonary arterial hypertension.

### Association of PAC and survival

With a median follow-up time until death or last follow-up of 5.1 years (IQR 4.1-6.0 years), a higher PAC was associated with lower hazard of death and lower hazard for the combined outcome of death or transplant (HR = 0.60 per 1mL/mmHg better compliance, 95% CI [0.46, 0.79], *p* < 0.001 and HR = 0.62 per 1mL/mmHg better compliance, 95% CI [0.49, 0.79], *p* < 0.001, respectively) (Table 4). This relationship of increased PAC and improved long-term outcomes was essentially unchanged after accounting for PVR, suggesting that there was an independent relationship between PAC and mortality that was not dependent on resistive afterload. Interestingly, the relationship between compliance and death or transplant was markedly attenuated with adjustment by mPAP (Supplemental Table 5). Further post-hoc exploratory analyses after observing this result were stratified around a median mPAP of 40 mmHg (p-value for the interaction = 0.001). In participants with a mPAP ≤ 40mmHg there was a lower hazard of death with improved compliance (HR = 0.44 per 1 mL/mmHg better compliance [95% CI 0.24, 0.82], *p* = 0.009) but no significant relationship between compliance and death when the mPAP > 40 mmHg (HR 1.26 per 1 mL/mmHg better compliance [95% CI 0.83, 1.89] *p* = 0.27). This suggests that increased PAC was associated with improved outcomes in the group with a lower mPAP but not in the group with higher mPAP, raising the possibility that there may be an isobaric threshold beyond which differences in compliance may not be associated with improved outcomes.

**Table 4:** In Patients With PAH, Death or Transplant Relative to Pulmonary Arterial Compliance Accounting for Differences in Body Habitus (Height and Weight), Age, Sex, and Pulmonary Vascular Resistance (PVR) in Staged Models (*n* = 328)

	Hazard of death per 1 mL/mmHg better compliance		
	HR	95% CI	*p*-value
Death			
Unadjusted	0.63	(0.49, 0.80)	<0.001
Adjusted for body habitus (height and weight)	0.60	(0.46, 0.77)	<0.001
Adjusted for age, sex, PAH etiology, and body habitus	0.60	(0.46, 0.79)	<0.001
Adjusted for age, sex, PAH etiology, body habitus, and PVR	0.54	(0.36, 0.79)	0.002
			
Death or Transplant			
Unadjusted	0.63	(0.51, 0.79)	<0.001
Adjusted for body habitus (height and weight)	0.62	(0.49, 0.78)	<0.001
Adjusted for age, sex, PAH etiology, and body habitus	0.62	(0.49, 0.79)	<0.001
Adjusted for age, sex, PAH etiology, body habitus, and PVR	0.53	(0.37, 0.76)	<0.001

PAH, Pulmonary arterial hypertension.

## Discussion

In this analysis of the large, multicenter PVDOMICS cohort, we provide nuanced insights into PAC and its phenotypic factors, report a novel association with exercise performance, and explore its pressure-dependent relationship with survival. We also reinforce a body of literature suggesting that compliance and resistance may not have a fixed relationship.^13^, ^20^, ^21^, ^22^ Patient-level factors such as age, height, time since diagnosis, PAWP, and the presence of connective tissue disease had independent associations with PAC. While relationships with cardiac morphology were not independent of PVR, relationships between PAC, exercise performance, and survival persisted even among individuals of otherwise similar PVR.

Prior studies of the relationship between patient-level factors and compliance showed a decrease in RC time with aging.^13^, ^23^, ^24^ We similarly found that older age is associated with worse PAC, which persisted even among individuals with similar PVR. In addition, we found that a higher PAWP was associated with worse compliance even among individuals with similar PVR. This corroborates and reinforces the work by Tedford and colleagues who showed elevation of PAWP negatively correlates with RC time, thus impacting RV pulsatile load.^13^

This study deepens our phenotypic understanding of PAC across PAH etiologies. The finding that individuals with CTD-PAH had worse compliance may be particularly important when paired with the observation that prognosis for CTD-PAH is poor relative to other etiologies.^25^, ^26^, ^27^, ^28^, ^29^ This raises several questions. A previous cohort similarly found that individuals with CTD-PAH had lower compliance; however, in that analysis (focused on RC time), the relationship appeared to be explained by confounders (age and mPAP).^22^ In contrast, we did not find compelling evidence that confounding explained the relationship. While there is evidence that poor outcomes in CTD-PAH may be mediated by differences in cardiac adaptation, differences in pulsatile afterload and a paradigm of “unmeasured load” is a complementary hypothesis that may help explain poor outcomes.^30^ It is possible that histopathologic changes of vasculitis and/or fibrosis could contribute to stiffness across the pulmonary vascular bed for a given resistance, although additional imaging or biomarker studies for vascular inflammation would be warranted to test this hypothesis.^31^ Previous evidence argued that differences in compliance among individuals with CTD-PAH were explained by other factors and, while we only identify small differences in compliance at a population-level, these PVDOMICS results may reinvigorate this debate.^22^

Compliance was associated with some aspects of CMR-derived morphology. This is physiologically intuitive, as compliance is highly correlated with overall right heart afterload.^7^ Interestingly, none of the associations with cardiac morphology at rest persisted after adjustment by PVR. Although surprising and different than other outcomes, this suggests that pulsatile afterload does not have an independent contribution to resting cardiac morphology beyond that quantified by PVR. This agrees with work by Caravita and colleagues to cautiously suggest that static right heart afterload (estimated by PVR) rather than compliance may be particularly influential in RV remodeling and aligns with the observations by Simpson and colleagues who found that ventricular mass index as measured by CMR had a linear relationship with PVR.^32^, ^33^

This study provides novel and previously unreported evidence that PAC may be a key factor in exercise performance in PH. We found a positive association between PAC and exercise capacity as measured by maximal oxygen consumption, watts, and METs even when adjusted for PVR. Although an independent relationship between compliance and exercise among individuals of similar PVR has not been previously reported, it comports with our understanding of exercise physiology. Exercise is a period of augmented pulsatility, and a disproportionate burden of low compliance may exacerbate poor RV-PA coupling during activity. This corroborates prior studies that suggest that PAH patients with preserved RV-PA coupling walk further; additionally, CMR-derived PAC estimates in left heart failure have found an association between PAC and functional capacity.^34^, ^35^, ^36^ It is possible that adequate compliance, alongside sufficient RV contractile reserve, mediates RV-PA coupling, which may also contribute to the association seen between PAC and survival.^34^, ^37^, ^38^

Our work focused on PAC among individuals with otherwise similar PVR. We join others to suggest that PAC cannot be uniformly predicted by PVR. A prior study that also highlighted variable RC-time suggested that isobaric PAH evaluation focused on PA pressure itself may provide an alternative perspective to that inferred when focusing on PVR adjustment. This approach acknowledges the pressure dependence of pulmonary arterial stiffness.^14^ In our study, accounting for differences in mPAP did not have a strong impact on the relationships between compliance, patient-level factors, CMR, and exercise but did impact the otherwise strong independent association of PAC with mortality. Our exploratory results suggest a strong association between compliance and death among those with low mPAP but did not find a similar association among those with high mPAP. This reinforces a potential framework whereby differences in compliance may be particularly relevant among individuals with lower PA pressures and may not be independently relevant at high pressures.^39^ In other words, there may be a pressure threshold beyond which PAC lacks an independent association with outcomes. This is a novel and hypothesis-generating result. Future work exploring the role of PAC in prognostic modeling may focus on the lower end of the PA pressure spectrum, especially with the current push to focus on pulmonary vascular disease at thresholds of mPAP.^40^

Limitations of this study include the use of a compliance surrogate. Although PAC approximation by SV/PApp is accepted, it may overestimate the total arterial compliance and is one of many approaches to estimate compliance.^41^, ^42^ While we used PAWP as a surrogate for left-heart disease and participants were adjudicated as having PAH, more nuanced parameters such as diastolic metrics and left atrial volume index would deepen our understanding of the relationship to cardiac comorbidities and the possibility that occult heart failure with preserved ejection fraction may have contributed to both reduced compliance and poor outcomes. Importantly, this study remains observational, and while we endeavored to account for confounding, unmeasured and residual confounding in observed relationships are legitimate concerns. Although many of our results reinforced previous observations, some did not. In particular, the paradigm of unmeasured load described by PAC in individuals with CTD-PAH contrasts with previous observations, is hypothesis-generating, and deserves further scrutiny.^22^ Importantly, the relationships described are complex, cause and effect are unclear, and inference should be cautious. For example, we report an association between heart rate and PAC. While there is evidence to suggest tachycardia reduces PAC, it is also possible that poor compliance could limit SV and lead to high heart rates.^20^ Relatedly, it is reasonable to wonder whether significant associations were driven by SV alone in this composite variable. In exploratory analyses, relationships with SV were weaker and -in contrast to PAC- were no longer significant after accounting for PVR (Supplemental Tables 6 and 7). Nevertheless, we remain cautious about speculating too heavily about the complex interplay between compliance, afterload writ large, myocardial vulnerability, heart rate, and SV outside of an experimental model. The association of longer time since diagnosis with improved compliance may reflect survivorship bias. In addition, while differences stratified by a range of characteristics were small at a population level, variation at the individual level was larger, and our work suggests that even modest differences in compliance may have clinical relevance. Of note CMR and exercise testing were advanced phenotyping elements of the protocol and not performed for all participants. Some differences between exercise and imaging results may reflect subtle differences in the included participants. Finally, while results were broadly reinforcing and argue against false detection to explain the bulk of the findings, there were several comparisons in this study which increases the possibility that some associations may have occurred by chance.

In conclusion, this study of the PVDOMICS cohort reinforces patient-level phenotypic factors associated with worse PAC. Differences in compliance were associated with exercise performance, mortality, and transplant even among individuals with similar PVR. Patients with PAH who have worse compliance have a distinct prognosis, physiology, and exercise capacity; this population provides an opportunity to deepen our understanding of PAH risk stratification, endotypes of disease, and therapeutic options.

## Financial support

The study received grants U01 HL125218 (Principal Investigator: Dr Rosenzweig), U01 HL125205 (Principal Investigator: Dr Frantz), U01 HL125212 (Principal Investigator: Dr Hemnes), U01 HL125208 (Principal Investigator: Dr Rischard), U01 HL125175 (Principal Investigator: Dr Hassoun), U01 HL125215 (Principal Investigator: Dr Leopold), and U01 HL125177 (Principal Investigator: Dr Beck) and was supported by the Pulmonary Hypertension Association. Unrelated to this work, Dr Mukherjee serves on the DSMB for Advarra, Inc and as a consultant for All Rock Bio. She receives funding from the NIH/NHLBI R01HL162851 and the US Department of Defense PR231648. Dr Leary has received grants and research support from the NHLBI, Bayer, and Janssen. He has been a grant review consultant for Bayer and has adjudicated clinical endpoints for Sumitomo Pharma. He is on the scientific leadership council for the Pulmonary Hypertension Association and the medical advisory board for Team PHenomenal Hope.

## Author Contributions

P.L., H.P., and L.O. conceived the study. Statistical analysis was performed by P.L. and J.R., T.W. B.H., W.W.H.T., E.M.H., and M.M. contributed to the interpretation of results. T.W. took the lead in writing the manuscript. All authors provided critical feedback, influenced the direction of the research, analysis, and manuscript.

## Declaration of Generative AI and AI-Assisted Technologies in the Writing Process

The authors used ChatGPT (OpenAI) to assist with language editing and clarity of expression. All content was reviewed and verified by the authors, who take full responsibility for the final manuscript.

## Conflicts of Interest statement

The authors declare the following financial interests/personal relationships, which may be considered as potential competing interests: PVDOMICS reports financial support was provided by the National Heart Lung and Blood Institute. Mukherjee reports a relationship with Advarra Inc that includes: consulting or advisory. Mukherjee reports a relationship with All Rock Bio that includes: consulting or advisory. Leary reports a relationship with Bayer Corporation that includes: funding grants. Leary reports a relationship with Janssen Pharmaceuticals Inc that includes: non-financial support. Leary reports a relationship with Sumitomo Pharma Co. Ltd., that includes: consulting or advisory. Leary reports a relationship with Pulmonary Hypertension Association that includes: board membership. Leary reports a relationship with Team PHenomenal Hope that includes: board membership. If there are other authors, they declare that they have no known competing financial interests or personal relationships that could have appeared to influence the work reported in this paper.

## Footnotes

Appendix A Supplementary data associated with this article can be found in the online version at doi:10.1016/j.jhlto.2026.100560.
