# Supplementary material for: Vascular compliance phenotyping in pulmonary arterial hypertension (a PVDOMICS study)
Source: JHLT Open. 2026 Apr 11;13:100560. doi: 10.1016/j.jhlto.2026.100560 (PMC13156711; doi:10.1016/j.jhlto.2026.100560)
Supplement: Supplementary file 1 — Supplementary material [file mmc1.docx]

**Title:** Pulmonary Compliance in Pulmonary Arterial Hypertension: A Study of the PVDOMICS Cohort

**Authors:** Talitha G. Wilson, MD; Laura Oppegard, MD; Hongyang Pi, MD MS; Brian A. Houston, MD; W.H. Wilson Tang, MD, Evelyn M. Horn, MD, Monica Mukherjee, MD MPH, Peter J. Leary, MD PhD; Jeff Robinson, MD

**Online supplement of exploratory analyses including isobaric evaluation and stroke volume**

| **Supplemental Table 1.** The difference in compliance for individuals with connective tissue disease associated PAH relative to idiopathic PAH when considering a range of exploratory adjustments for potential confounders in this relationship (n=252). | | | |
| --- | --- | --- | --- |
|  | Difference in compliance (mL/mmHg) | | |
|  | β | 95% CI | p-value |
| Connective tissue disease PAH relative to idiopathic PAH |  |  |  |
| Unadjusted | -0.52 | (-0.96, -0.08) | 0.02 |
| Adjusted for pulmonary vascular resistance | -0.38 | (-0.72, -0.03) | 0.03 |
| Adjusted for PVR and time since diagnosis | -0.34 | (-0.69, 0.00) | 0.05 |
| Adjusted for PVR and wedge pressure | -0.42 | (-0.77, -0.08) | 0.02 |
| Adjusted for PVR and mPA pressure | -0.54 | (-0.87, -0.21) | 0.001 |
| Adjusted for PVR and heart rate | -0.37 | (-072, -0.02) | 0.04 |
| Adjusted for ALL covariates | -0.38 | (-0.71, -0.04) | 0.03 |
| *Of note, this is a limited cohort for an exploratory analysis comprised of only idiopathic PAH and connective tissue disease associated PAH. As such, the overall number of included participants, point estimates, confidence interval, and p-value differs from Table 1 in the main manuscript where all participants with PAH were included. | | | |

| **Supplemental Table 2**. In patients with PAH, difference in pulmonary arterial compliance by participant characteristic in unadjusted models, among individuals of otherwise similar mean pulmonary artery pressure (mPAP) as evaluated by adjustment (negative compliance is worse compliance and positive compliance is better compliance) (n=328). | | | | | | |  |
| --- | --- | --- | --- | --- | --- | --- | --- |
|  | Difference in Compliance | | | Difference in Compliance | | | |
|  | (unadjusted) | | | (individuals with similar mPAP) | | | |
|  | β | 95% CI | p-value | β | 95% CI | p-value | |
| Age (per 10 years) | -0.14 | (-0.26, -0.02) | 0.02 | -0.26 | (-0.36, -0.17) | <0.001 | |
| Female | -0.07 | (-0.46, 0.32) | 0.72 | -0.30 | (-0.61, 0.01) | 0.06 | |
| Height (per 10 cm) | 0.24 | (0.07, 0.42) | 0.006 | 0.26 | (0.13, 0.40) | <0.001 | |
| BMI (per 5kg/m^2^) | 0.08 | (-0.04, 0.19) | 0.19 | 0.08 | (-0.01, 0.17) | 0.10 | |
| Time since diagnosis (per 3 years) | 0.13 | (0.04, 0.22) | 0.003 | 0.14 | (0.07, 0.21) | <0.001 | |
| Heart rate (per 10 beats/minute) | -0.26 | (-0.38, -0.13) | <0.001 | -0.05 | (-0.16, 0.06) | 0.39 | |
| PAWP (per 1mmHg) | -0.04 | (-0.07, -0.01) | 0.01 | 0.4 | (0.01, 0.07) | 0.003 | |
| History of Hypertension | -0.27 | (-0.64, 0.09) | 0.14 | -0.37 | (-0.66, -0.09) | 0.01 | |
| History of Diabetes | -0.07 | (-0.52, 0.39) | 0.77 | -0.14 | (-0.50, 0.22) | 0.45 | |
| PAH Etiology |  |  |  |  |  |  | |
| Idiopathic |  | Referent |  |  | Referent |  | |
| Drug/Toxin | -0.57 | (-1.42, 0.28) | 0.19 | -0.43 | (-1.08, 0.22) | 0.20 | |
| Connective Tissue Disease | -0.52 | (-0.94, -0.11) | 0.01 | -0.63 | (-0.95, -0.31) | <0.001 | |
| Other | -0.31 | (-0.87, 0.26) | 0.29 | -0.02 | (-0.38, 0.34) | 0.91 | |

| **Supplemental Table 3.** In patients with PAH, difference in cardiac morphology relative to pulmonary arterial compliance accounting for differences in body habitus (height and weight), age, sex, and mean pulmonary artery pressure (mPAP) in staged models (n=225). | | | |
| --- | --- | --- | --- |
|  | Difference in Outcome per 1mL/mmHg better compliance | | |
|  | β | 95% CI | p-value |
| RV-EDV (mL) |  |  |  |
| Unadjusted | -5.2 | (-11.8, 1.4) | 0.12 |
| Adjusted for body habitus (height and weight) | -7.1 | (-13.3, -0.9) | 0.03 |
| Adjusted for age, sex, PAH etiology, and body habitus | -7.3 | (-13.6, -1.0) | 0.02 |
| Adjusted for age, sex, PAH etiology, body habitus, and mPAP | 2.5 | (-5.3, 10.4) | 0.53 |
|  |  |  |  |
| RA-EDV (mL) |  |  |  |
| Unadjusted | -4.7 | (-7.3, -2.0) | 0.001 |
| Adjusted for body habitus (height and weight) | -5.2 | (-7.8, -2.6) | <0.001 |
| Adjusted for age, sex, PAH etiology, and body habitus | -5.1 | (-7.7, -2.4) | <0.001 |
| Adjusted for age, sex, PAH etiology, body habitus, and mPAP | -1.0 | (-4.3, 2.3) | 0.56 |
|  |  |  |  |
| RV-Mass (g) |  |  |  |
| Unadjusted | -1.7 | (-3.2, -0.1) | 0.04 |
| Adjusted for body habitus (height and weight) | -2.1 | (-3.6, -0.6) | 0.005 |
| Adjusted for age, sex, PAH etiology, and body habitus | -2.1 | (-3.6, -0.6) | 0.005 |
| Adjusted for age, sex, PAH etiology, body habitus, and mPAP | 1.3 | (-0.5, 3.1) | 0.15 |
|  |  |  |  |
| RV-EF (%) |  |  |  |
| Unadjusted | 2.7% | (1.8%, 3.6%) | <0.001 |
| Adjusted for body habitus (height and weight) | 2.9% | (2.0%, 3.7%) | <0.001 |
| Adjusted for age, sex, PAH etiology, and body habitus | 2.9% | (2.0%, 3.9%) | <0.001 |
| Adjusted for age, sex, PAH etiology, body habitus, and mPAP | 0.4% | (-0.6%, 1.5%) | 0.45 |

| **Supplemental Table 4.**  In patients with PAH, difference in exercise and the diffusing capacity of the lung for carbon monoxide (DLCO) relative to pulmonary arterial compliance accounting for differences in body habitus (height and weight), age, sex, and mean pulmonary artery pressure (mPAP) in staged models. | | | |
| --- | --- | --- | --- |
|  | Difference in Outcome per 1mL/mmHg better compliance | | |
|  | β | 95% CI | p-value |
| Maximal Oxygen Consumption (mL/kg/min) (n=236) |  |  |  |
| Unadjusted | 1.2 | (0.9, 1.5) | <0.001 |
| Adjusted for height (oxygen consumption already indexed to weight) | 1.1 | (0.8, 1.4) | <0.001 |
| Adjusted for age, sex, PAH etiology, and height | 1.1 | (0.8, 1.4) | <0.001 |
| Adjusted for age, sex, PAH etiology, height, and mPAP | 0.8 | (0.4, 1.2) | <0.001 |
|  |  |  |  |
| Maximal Watts (n=236) |  |  |  |
| Unadjusted | 9.4 | (6.6, 12.1) | <0.001 |
| Adjusted for body habitus (height and weight) | 8.1 | (5.4, 10.8) | <0.001 |
| Adjusted for age, sex, PAH etiology, and body habitus | 7.4 | (4.8, 9.7) | <0.001 |
| Adjusted for age, sex, PAH etiology, body habitus, and mPAP | 4.4 | (1.2, 7.7) | 0.008 |
|  |  |  |  |
| Maximal Mets (n=236) |  |  |  |
| Unadjusted | 0.3 | (0.2, 0.4) | <0.001 |
| Adjusted for body habitus (height and weight) | 0.3 | (0.2, 0.4) | <0.001 |
| Adjusted for age, sex, PAH etiology, and body habitus | 0.3 | (0.2, 0.4) | <0.001 |
| Adjusted for age, sex, PAH etiology, body habitus, and mPAP | 0.2 | (0.1, 0.3) | <0.001 |
|  |  |  |  |
| DLCO (n=87) |  |  |  |
| Unadjusted | 1.1 | (0.3, 1.9) | 0.006 |
| Adjusted for body habitus (height and weight) | 0.3 | (-0.4, 1.1) | 0.35 |
| Adjusted for age, sex, PAH etiology, and body habitus | 0.4 | (-0.2, 1.0) | 0.15 |
| Adjusted for age, sex, PAH etiology, body habitus, and mPAP | 0.5 | (-0.4, 1.3) | 0.26 |

| **Supplemental Table 5.** In patients with PAH, death or transplant relative to pulmonary arterial compliance accounting for differences in body habitus (height and weight), age, sex, and mean pulmonary artery pressure (mPAP) in staged models (n=328). | | | |
| --- | --- | --- | --- |
|  | Hazard of death per 1mL/mmHg better compliance | | |
|  | HR | 95% CI | p-value |
| Death |  |  |  |
| Unadjusted | 0.63 | (0.49, 0.80) | <0.001 |
| Adjusted for body habitus (height and weight) | 0.60 | (0.46, 0.77) | <0.001 |
| Adjusted for age, sex, PAH etiology, and body habitus | 0.60 | (0.46, 0.79) | <0.001 |
| Adjusted for age, sex, PAH etiology, body habitus, and mPAP | 0.85 | (0.61, 1.20) | 0.37 |
|  |  |  |  |
| Death or Transplant |  |  |  |
| Unadjusted | 0.63 | (0.51, 0.79) | <0.001 |
| Adjusted for body habitus (height and weight) | 0.62 | (0.49, 0.78) | <0.001 |
| Adjusted for age, sex, PAH etiology, and body habitus | 0.62 | (0.49, 0.79) | <0.001 |
| Adjusted for age, sex, PAH etiology, body habitus, and mPAP | 0.88 | (0.65, 1.17) | 0.38 |

| **Supplemental Table 6.** In patients with PAH, difference exercise and the diffusing capacity of the lungs for carbon monoxide (DLCO) relative to stroke volume accounting for differences in body habitus (height and weight), age, sex, and pulmonary vascular resistance (PVR) in staged models (n=236). | | | |  |
| --- | --- | --- | --- | --- |
|  | Difference in Outcome per  5 mL larger stroke volume | | | |
|  | β | 95% CI | p-value | |
| Maximal Oxygen Consumption (mL/kg/min) |  |  |  | |
| Unadjusted | 0.2 | (0.1, 0.3) | <0.001 | |
| Adjusted for body height (consumption already indexed to weight) | 0.2 | (0.1, 0.3) | 0.001 | |
| Adjusted for age, sex, PAH etiology, and height | 0.2 | (0.1, 0.3) | 0.003 | |
| Adjusted for age, sex, PAH etiology, height, and PVR | 0.0 | (-0.1, 0.2) | 0.70 | |
|  |  |  |  | |
| Maximal Watts |  |  |  | |
| Unadjusted | 2.5 | (1.5, 3.5) | <0.001 | |
| Adjusted for body habitus (height and weight) | 1.8 | (0.8, 2.8) | <0.001 | |
| Adjusted for age, sex, PAH etiology, and body habitus | 1.6 | (0.7, 2.5) | 0.001 | |
| Adjusted for age, sex, PAH etiology, body habitus, and PVR | 0.4 | (-0.7, 1.6) | 0.45 | |
|  |  |  |  | |
| Maximal Mets |  |  |  | |
| Unadjusted | 0.1 | (0.0, 0.1) | <0.001 | |
| Adjusted for body habitus (height and weight) | 0.1 | (0.0, 0.1) | <0.001 | |
| Adjusted for age, sex, PAH etiology, and body habitus | 0.1 | (0.0, 0.1) | <0.001 | |
| Adjusted for age, sex, PAH etiology, body habitus, and PVR | 0.0 | (0.0, 0.1) | 0.36 | |

| **Supplemental Table 7.** In patients with PAH, death or transplant relative to stroke volume accounting for differences in body habitus (height and weight), age, sex, and pulmonary vascular resistance (PVR) in staged models (n=328). | | | | |
| --- | --- | --- | --- | --- |
|  | Hazard of death per  5mL larger stroke volume | | |  |
|  | HR | 95% CI | p-value |  |
| Death |  |  |  |  |
| Unadjusted | 0.96 | (0.92, 1.01) | 0.09 |  |
| Adjusted for body habitus (height and weight) | 0.95 | (0.90, 0.99) | 0.03 |  |
| Adjusted for age, sex, PAH etiology, and body habitus | 0.96 | (0.91, 1.01) | 0.12 |  |
| Adjusted for age, sex, PAH etiology, body habitus, and PVR | 1.00 | (0.93, 1.06) | 0.91 |  |
|  |  |  |  |  |
| Death or Transplant |  |  |  |  |
| Unadjusted | 0.96 | (0.92, 1.00) | 0.09 |  |
| Adjusted for body habitus (height and weight) | 0.96 | (0.92, 1.00) | 0.06 |  |
| Adjusted for age, sex, PAH etiology, and body habitus | 0.97 | (0.92, 1.01) | 0.16 |  |
| Adjusted for age, sex, PAH etiology, body habitus, and PVR | 1.00 | (0.94, 1.06) | 0.99 |  |
